# Identification of autophagy-related genes in osteoarthritis articular cartilage and their roles in immune infiltration

**DOI:** 10.3389/fimmu.2023.1263988

**Published:** 2023-11-27

**Authors:** Jun Qin, Jin Zhang, Jian-Jun Wu, Xiao Ru, Qiu-Ling Zhong, Jin-Min Zhao, Ni-Han Lan

**Affiliations:** ^1^ Guangxi Engineering Center in Biomedical Materials for Tissue and Organ Regeneration, The First Affiliated Hospital of Guangxi Medical University, Nanning, China; ^2^ Guangxi Clinical Medical Research Center for Orthopedic Disease, The First Affiliated Hospital of Guangxi Medical University, Nanning, China; ^3^ Department of Medical Cosmetology, The First Affiliated Hospital of Guangxi Medical University, Nanning, China; ^4^ Department of Orthopaedics Trauma and Hand Surgery, The First Affiliated Hospital of Guangxi Medical University, Nanning, China; ^5^ Department of Orthopedics, Zhanjiang Central Hospital, Guangdong Medical University, Zhanjiang, China; ^6^ Research Centre for Regenerative Medicine, Department of Orthopedics, The First Affiliated Hospital of Guangxi Medical University, Nanning, China

**Keywords:** osteoarthritis, cartilage, autophagy, immune cell infiltration, bioinformatics analysis

## Abstract

**Background:**

Autophagy plays a critical role in the progression of osteoarthritis (OA), mainly by regulating inflammatory and immune responses. However, the underlying mechanisms remain unclear. This study aimed to investigate the potential relevance of autophagy-related genes (ARGs) associated with infiltrating immune cells in OA.

**Methods:**

GSE114007, GSE169077, and ARGs were obtained from the Gene Expression Omnibus (GEO) database and the Human Autophagy database. R software was used to identify the differentially expressed autophagy-related genes (DEARGs) in OA. Functional enrichment and protein–protein interaction (PPI) analyses were performed to explore the role of DEARGs in OA cartilage, and then Cytoscape was utilized to screen hub ARGs. Single-sample gene set enrichment analysis (ssGSEA) was used to conduct immune infiltration analysis and evaluate the potential correlation of key ARGs and immune cell infiltration. Then, the expression levels of hub ARGs in OA were further verified by the GSE169077 and qRT-PCR. Finally, Western blotting and immunohistochemistry were used to validate the final hub ARGs.

**Results:**

A total of 24 downregulated genes and five upregulated genes were identified, and these genes were enriched in autophagy, mitophagy, and inflammation-related pathways. The intersection results identified nine hub genes, namely, CDKN1A, DDIT3, FOS, VEGFA, RELA, MAP1LC3B, MYC, HSPA5, and HSPA8. GSE169077 and qRT-PCR validation results showed that only four genes, CDKN1A, DDT3, MAP1LC3B, and MYC, were consistent with the bioinformatics analysis results. Western blotting and immunohistochemical (IHC) showed that the expression of these four genes was significantly downregulated in the OA group, which is consistent with the qPCR results. Immune infiltration correlation analysis indicated that DDIT3 was negatively correlated with immature dendritic cells in OA, and FOS was positively correlated with eosinophils.

**Conclusion:**

CDKN1A, DDIT3, MAP1LC3B, and MYC were identified as ARGs that were closely associated with immune infiltration in OA cartilage. Among them, DDIT3 showed a strong negative correlation with immature dendritic cells. This study found that the interaction between ARGs and immune cell infiltration may play a crucial role in the pathogenesis of OA; however, the specific interaction mechanism needs further research to be clarified. This study provides new insights to further understand the molecular mechanisms of immunity involved in the process of OA by autophagy.

## 1 Introduction

Osteoarthritis (OA) is a common chronic inflammatory disease in clinical practice, and its development is related to many factors including age, joint damage, and obesity (1). The development is characterized by inflammation, cartilage degeneration, narrowing of the joint space, formation of osteophytes, and sclerosis of the subchondral bone (2). Currently, there is no effective treatment to alleviate the disease. While non-steroidal anti-inflammatory drug (NSAID) interventions mainly address pain and inflammation, OA cannot be prevented. Joint replacement surgery is often a solution for end-stage patients but imposes a significant financial burden (3, 4). Therefore, understanding the molecular mechanisms of OA pathogenesis will provide new ideas for the treatment of OA. The process of OA has been reported to be associated with aging, inflammation, apoptosis, and autophagy (1, 5, 6). Previous studies have shown that the dysfunction of autophagy is a major factor in OA (7). Therefore, identifying novel molecular biological targets is crucial for the in-depth study of the underlying molecular mechanisms by which autophagy regulates the pathogenesis of OA.

Autophagy is an intracellular biological degradation system with highly evolutionary conservative features that slow down the OA process by restoring cellular dysfunction in cartilage (8). Autophagy has great clinical prospects as a potential therapeutic target for OA; however, the specific pathogenesis of OA exacerbated by dysregulated autophagy has not been elucidated. Previous research has indicated that mTOR, LC3-II, Beclin-1, and p62 were associated with OA progression and provide promising therapeutic targets for OA (9, 10). As an important key regulator, mTOR affects autophagic activity and is a key target for the autophagic pathway (8). Microtubule-associated proteins 1A/1B light chain 3B (LC3) are involved in the biosynthesis of autophagosomes. In OA, increased chondrocyte apoptosis was associated with the decrease of LC3 (11). The levels of autophagy-related markers, such as ATG5, LC3-II, and Beclin-1 gene expression, were significantly increased in chondrocytes in the early stage of OA. With the aggravation of the disease, oxidative stress-induced damage increased gradually while decreasing autophagy, resulting in chondrocyte hypertrophy (12). These research data demonstrate that autophagy plays a key role in OA. However, autophagy-related genes (ARGs) in OA development are still largely unknown. Therefore, in-depth exploration of autophagy-related markers in OA through bioinformatics could help identify new potential biomarkers for the treatment of osteoarthritis. In addition, studies have indicated that immune regulation may also play a key role in the pathogenesis of OA (13, 14). Autophagy is an important process in regulating immune responses, which not only eliminates infectious agents and modulates inflammatory responses but also selects antigen presentation and regulates T-cell homeostasis and activation (15, 16). Huang et al. showed that autophagy and immune regulation played different roles in OA and RA, but both are significantly correlated (17). However, few reports have comprehensively investigated the relationship between ARG expression and cartilage immune infiltration in OA and the role of ARGs’ biological functions in cartilage immune infiltration. Therefore, we hypothesized that ARGs were differentially expressed in OA and normal cartilage tissues, and the change of autophagy level may related to immune cell infiltration.

In this study, we identified ARGs associated with OA based on OA-related data in the Gene Expression Omnibus (GEO) database. We found the association of the hub genes including *CDKN1A*, *DDIT3*, *MAP1LC3B*, and *MYC* with immune cell infiltration in OA, which was further verified using qRT-PCR, Western blotting, and immunohistochemical staining. Our study hopes to identify ARGs in OA cartilage tissue and then determine whether they regulate OA by modulating immune cell infiltration, which will provide a novel perspective for OA pathogenesis.

## 2 Materials and methods

### 2.1 Acquisition of raw data and pre-processing

**Figure 1:**
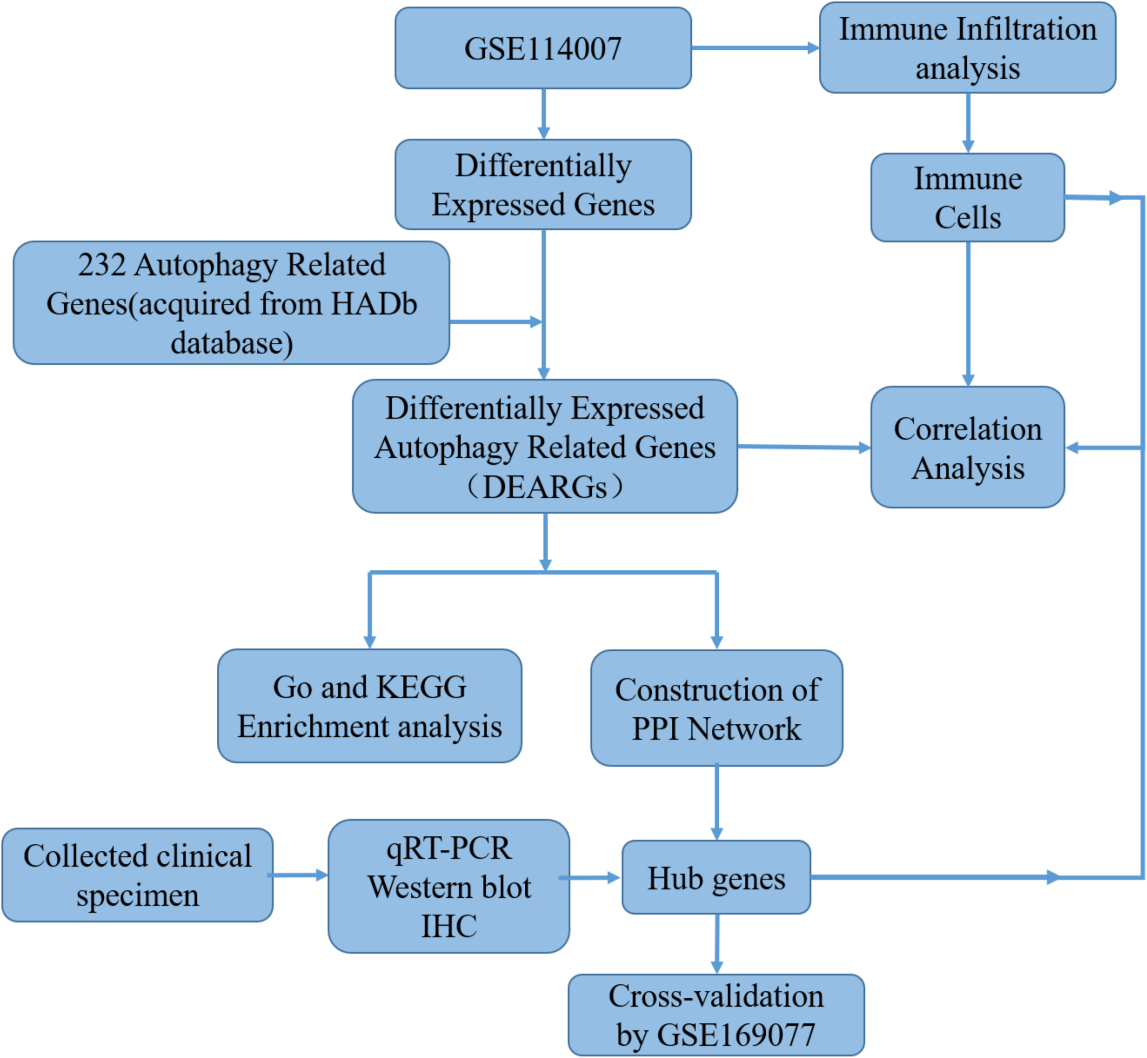
The workflow chart of this study.

Two independent human knee articular cartilage tissue mRNA expression profile datasets were downloaded from the GEO database (https://www.ncbi.nlm.nih.gov/geo/). The GSE114007 RNA-seq dataset included 20 OA samples and 18 normal samples from two different platforms, GPL18573 and GPL11154 (18). GSE169077 microarray dataset (GPL96 platform) included six OA samples and five normal samples, which served as a validation dataset for verifying hub genes. Detailed information on the datasets is shown in **Table S1**. In addition, 222 ARGs were obtained from the HADb database (http://www.autophagy.lu). The workflow of this research is shown in **Figure 1**.

### 2.2 Identification of differentially expressed autophagy-related genes

The GSE114007 gene expression of profile was quality assessed using the “factoextra” package (https://cloud.r-project.org/package=factoextra/) in R software before analyzing differentially expressed genes (DEGs) in OA, and sample clustering results suggested that there were large differences between two different platforms (**Supplementary Figure S1**); therefore, only data from the GPL18573 platform, which included 10 OA samples and 10 normal samples, were taken for subsequent analysis. The counts’ data were standardized by the transcripts per kilobase per million mapped reads (TPM), and then the DEGs between OA and normal knee cartilage tissue were screened using the R package “DESeq2” with the threshold set as |log2FoldChange| > 1 and *p*-value <0.05 (19). Subsequently, differentially expressed ARGs (DEARGs) were obtained by intersecting genes between DEGs and ARGs. The Venn diagram drawn through the “VennDiagram” package displayed the number of DEARGs (https://CRAN.R-project.org/package=VennDiagram). Heatmap, volcano plot, and boxplot were drawn through the “pheatmap”, “ggplot2”, and “ggpubr” packages of R software, respectively.

### 2.3 Functional enrichment analysis of DEARGs

The Gene Ontology (GO) analysis and Kyoto Encyclopedia of Genes and Genomes (KEGG) pathway enrichment of DEARGs were conducted in the R package “clusterProfiler” (20), and gene IDs were converted using Perl scripts. Molecular function (MF), biological process (BP), and cellular component (CC) constitute the GO annotation. Under the conditions of *p* < 0.05 and q < 0.05, GO terms and signaling pathways with significant differences were screened, and then the R software packages “enrichment plot”, “ggplot2”, and “GOplot” (21) were used to present the results, respectively.

### 2.4 Correlation analysis and PPI network analysis of DEARGs

The correlation between DEARG was investigated using Spearman’s correlation in the “Corrplot” package. The STRING online database (http://string-db.org/) was used to perform the protein–protein interaction (PPI) network analysis and investigate the underlying relationships between DEARGs with an interaction score >0.4, and then Cytoscape (version 3.8.1) was used to analyze and visualize the screened networks. MCODE and CytoHubba, two plug-ins of Cytoscape, use different algorithms to obtain autophagy-related hub genes. CytoHubba was used to calculate hub genes based on four different algorithms: maximal clique centrality (MCC), degree, closeness, and maximum neighborhood component (MNC). The top 10 genes were screened out of each algorithm as autophagy-related hub genes. Finally, the hub genes of DEARGs were defined as the overlapped genes obtained by five algorithms, and these genes were screened by drawing an UpSet diagram through the R package “UpSetR”.

### 2.5 Immune cell infiltration and correlation analysis

Single-sample gene set enrichment analysis (ssGSEA) was used to analyze the abundance of immune cell infiltration in OA and normal cartilage tissues and identify 28 types of immune cell infiltration (22). Immune cell infiltration between OA and normal samples was compared using the Wilcoxon test, and the results were then visualized by violin diagrams drawn through the “ggplots” package. The correlation analysis of 28 kinds of infiltrating immune cells was performed, and then the results were visualized using the “ggcorrplot” package. Finally, Spearman’s correlation analysis was used to analyze the correlation between hub ARGs and the infiltration degree of different immune cells, and the results were visualized using the “ggcorrplot” and “ggstatsplot” packages. Furthermore, CIBERSORT algorithm was also adopted to investigate the immune cell infiltration.(As shown in **Supplementary Figure S2**).

### 2.6 Validation of hub gene expression with other osteoarthritis datasets

To understand the expression of these hub ARGs in cartilage, validation was performed on the GSE169077 OA dataset using the “limma” package in R software. The heatmaps and boxplots were performed using the “pheatmap” and “ggplot2” packages in R language.

### 2.7 Clinical specimen collection and chondrocyte isolation and culture

To confirm the hub ARGs, knee cartilage tissue samples from five cases of OA and five cases of traumatic amputation were collected after obtaining written informed consent from all patients. The study was approved by the Ethics Committee of the First Affiliated Hospital of Guangxi Medical University (Nanning, China) and complied with the tenets of the Declaration of Helsinki.

The fresh cartilage tissues were rinsed with sterile phosphate-buffered saline (PBS) three times and then cut into the size of approximately 0.3–0.5-mm^3^ pieces. The tissues were first treated with trypsin/ethylenediaminetetraacetic acid (EDTA) (Solarbio, Beijing, China) at a concentration of 0.25% for 30 min and then digested with collagenase II (Solarbio, Beijing, China) at a concentration of 2 mg/mL for 4 h at 37°C. Dulbecco’s Modified Eagle Medium (DMEM; Gibco, Shanghai, China) was used to culture chondrocytes after isolation, which consisted of 10% (v/v) fetal bovine serum (FBS; Tianhang, Zhejiang, China) and 1% (v/v) antibiotics (penicillin 10,000 U/ml and streptomycin 10,000 μg/ml, Solarbio, Beijing, China), cultured at an incubator with 5% CO_2_ at 37°C. Passages 2–3 of chondrocytes were used for further experiments. Chondrocytes were divided into two groups: the control group (cultured with normal DMEM) and the treatment group (OA group). Chondrocytes treated with 10 ng/mL IL-1β (Solarbio, Beijing, China) for 24 hours were considered as the OA group. Then, total cellular RNA and protein were collected for further analysis.

### 2.8 RNA extraction and qRT-PCR

According to the instructions of HiPure Total RNA Mini Kit (Magen, Guangzhou, China), total RNA was extracted from normal and OA group chondrocytes, and then reverse transcription with a Reverse Transcription kit (Takara, Dalian, China) was performed after checking the RNA concentration by a micro-spectrophotometer (Thermo Fisher Scientific, Waltham, MA, USA). The mRNA expression levels of the DNA damage-inducible transcript 3 (*DDIT3*), cyclin-dependent kinase inhibitor 1A (*CDKN1A*), fos proto-oncogene (*FOS*), vascular endothelial growth factor A (*VEGFA*), rela proto-oncogene (*RELA*), heat shock protein family A member 5 (*HSPA5*), microtubule-associated protein 1 light chain 3 beta (*MAP1LC3B*), MYC proto-oncogene (*MYC*), and heat shock protein family A member 8 (*HSPA8*) were analyzed by qRT-PCR. **Table 1** shows the primer sequences for the main qRT-PCR used in this study. qRT-PCR was performed as previously reported (23).

**Table 1:** Primer sequences used in the qRT-PCR experiments.

Gene	Forward primer (5′–3′)	Reverse primer (3′–5′)
GAPDH	GTCAAGGCTGAGAACGGGAA	AAATGAGCCCCAGCCTTCTC
CDKN1A	CCCGTGAGCGATGGAACT	CCCGTGGGAAGGTAGAGC
DDIT3	ACCAGGAAACGGAAACAG	ACCATTCGGTCAATCAGA
FOS	ACCAGGAAACGGAAACAG	ACCATTCGGTCAATCAGA
RELA	AGAGCAGCGTGGGGACTA	ATGGGATGAGAAAGGACAGG
MAP1LC3B	CAGCATCCAACCAAAATC	CTGTAAGCGCCTTCTAAT
MYC	ATCCTGTCCGTCCAAGCA	CGCACAAGAGTTCCGTAG
HSPA5	TTGCCGTTCAAGGTGGTT	AGCGGTTTCTTTCATTTTAG
HSPA8	GACAACCGAATGGTCAAC	GTACGGAGGCGTCTTACA

### 2.9 Protein extraction and Western blotting

According to the instructions of RIPA Lysis Buffer (Beyotime, Shanghai, China), total protein was extracted from normal and OA chondrocytes, and then the concentration of the extracted protein was determined by bicinchoninic acid (BCA) protein detection kit (Beyotime, Shanghai, China). The protein samples (40 μg/lane) were then separated by 10% sodium dodecyl sulfate (SDS)–polyacrylamide gels, and the blots were incubated overnight using antibodies against CDKN1A (1:1,000; Beyotime), DDIT3 (1:1,000; Beyotime), MAP1LC3B (1:1,000; Proteintech, Chicago, IL, USA), MYC (1:2,000; Proteintech), and GAPDH (1:5,000; Sangon Biotech, Shanghai, China) at 4°C. After washing with TBST, the membranes were incubated with goat anti-rabbit horseradish peroxidase (HRP)-conjugated secondary antibody (1:5,000; Sangon Biotech) at room temperature for 1 hour. The protein blots were detected with an enhanced chemiluminescence (ECL) system (Beyotime, Shanghai, China), and images were acquired with the Amersham Imager (Cytiva, Uppsala, Sweden). The intensity of these blots was quantified using ImageJ software (NIH, Bethesda, MD, USA).

### 2.10 Immunohistochemical analysis

Immunohistochemical (IHC) staining was conducted using a universal two-step detection kit (PV-9000; ZSGB-BIO, Beijing, China) following the manufacturer’s instructions. After being dewaxed, the knee articular cartilage tissue slices were placed in EDTA antigen repair solution at 95°C for 15 min for antigen repair. Endogenous peroxidase’s activity was blocked with peroxidase blocking reagent for 10 min at room temperature (RT). An appropriate amount of rabbit anti-CDKN1A (1:200; Beyotime), rabbit anti-DDIT3 (1:200; Beyotime), rabbit anti-MAP1LC3B (1:200, Proteintech), and rabbit anti-MYC (1:200, Proteintech) primary antibody working solution was added dropwise on the tissue slices and then incubated in a wet box overnight at 4°C. After rinsing with PBS, the appropriate amount of goat anti-mouse/rabbit IgG polymer labeled with enhanced enzyme was added dropwise on tissue slices and incubated at 37°C for 20 min. Subsequently, the tissue slices were visualized with DAB staining, and then counterstaining was achieved with hematoxylin.

### 2.11 Statistical analysis

Data are expressed as mean ± standard deviation (SD). Gene expression or protein levels in the two groups of chondrocytes were compared by unpaired Student’s t-test or Wilcoxon test. R software (version 4.1.3) and GraphPad Prism 8.0 (San Diego, CA, USA) were used for statistical analysis and plots. All correlation analyses were performed using Spearman’s method. Statistical significance required *p* < 0.05 (two-sided).

## 3 Results

### 3.1 Differential expression of ARGs in osteoarthritis cartilage

**Figure 2:**
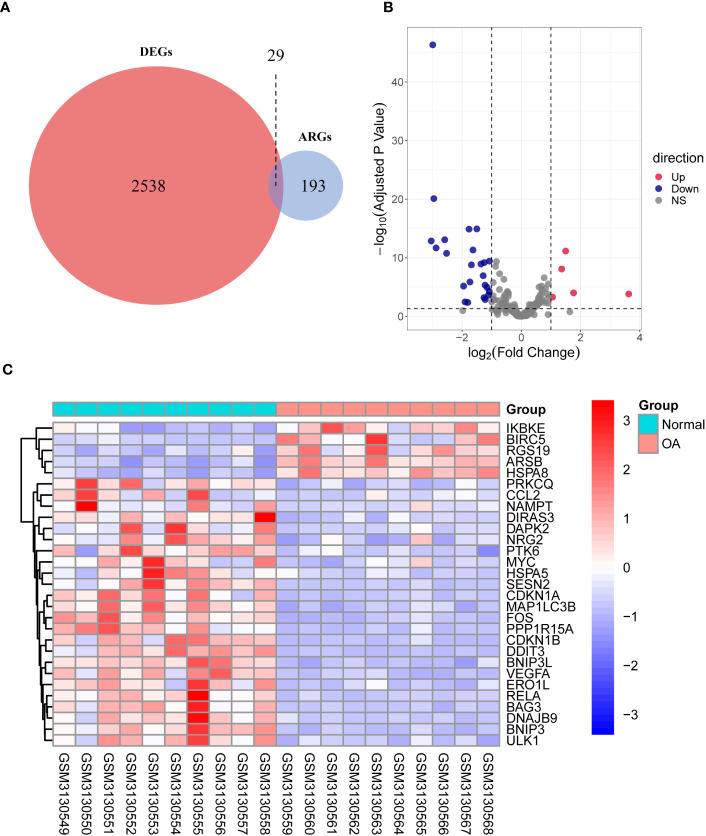
Identification of DEARGs. **(A)** Veen diagram showing the DEARGs. **(B)** Volcano plot displaying significantly differentially expressed ARGs. Red dots represent the upregulated genes, and dark purple dots denote the downregulated genes, with thresholds of |log2FoldChange| ≥ 1 and adjusted *p*-value <0.05. **(C)** The expressions of 29 DEARGs in OA are displayed in the heatmap. Red signifies significantly upregulated ARGs, and purple indicates significantly downregulated ARGs in the samples. DEARGs, differentially expressed autophagy-related genes; ARGs, autophagy-related genes; OA, osteoarthritis.

**Figure 3:**
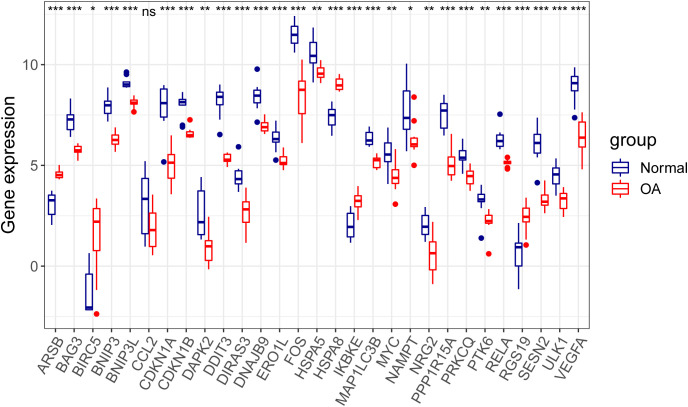
Boxplot displaying differential expressed of 29 autophagy-related genes in OA and normal cartilage samples. *p*-Values were calculated using Wilcoxon test. *p < 0.05; **p < 0.01; ***p < 0.01. ns, not significant; OA, osteoarthritis.

Through differential expression analysis of GPL18573 platform-derived data in the GSE114007 dataset, 2,567 genes were significantly expressed in OA compared with control cartilage tissue based on |log2Fold Change| > 1 with *p* < 0.05. Twenty-nine DEARGs were obtained from 222 ARGs, which intersected with 2,567 DEGs, and five upregulated genes and 24 downregulated genes were screened (**Figure 2A**, **Table 2**). Volcano plot and heatmap were used to visualize the expressions of the 29 DEARGs (**Figures 2B, C**). Furthermore, the boxplot showed 29 DEARG expression patterns in OA compared to control cartilage tissues (**Figure 3**).

**Table 2:** The 29 DEARGs in OA cartilage tissues compared to healthy control.

Gene symbol	log2FC	*p*-Value	Adj.*p*-value	Regulation
CDKN1A	−3.04249	1.18E−15	1.38E−13	Down
DDIT3	−2.99043	5.31E−51	4.64E−47	Down
SESN2	−2.95254	1.92E−23	8.17E−21	Down
FOS	−2.87791	2.31E−14	2.12E−12	Down
PPP1R15A	−2.58655	6.77E−16	8.51E−14	Down
VEGFA	−2.52378	2.48E−13	1.76E−11	Down
DAPK2	−1.94863	5.07E−07	6.87E−06	Down
NAMPT	−1.89816	0.000754	0.003466	Down
CCL2	−1.80804	0.000996	0.004336	Down
BNIP3	−1.76472	7.53E−18	1.31E−15	Down
DIRAS3	−1.73374	8.05E−08	1.37E−06	Down
DNAJB9	−1.68479	4.00E−11	1.64E−09	Down
BAG3	−1.63198	5.76E−14	4.79E−12	Down
CDKN1B	−1.50153	6.82E−18	1.20E−15	Down
RELA	−1.3636	2.77E−11	1.18E−09	Down
ULK1	−1.28682	4.83E−09	1.16E−07	Down
NRG2	−1.26045	0.000102	0.000644	Down
MAP1LC3B	−1.2514	1.44E−11	6.73E−10	Down
ERO1L	−1.22436	3.31E−07	4.75E−06	Down
MYC	−1.21702	0.000244	0.001345	Down
PRKCQ	−1.15477	8.88E−07	1.13E−05	Down
HSPA5	−1.10649	3.21E−05	0.000239	Down
PTK6	−1.08306	5.67E−06	5.50E−05	Down
BNIP3L	−1.07546	7.52E−12	3.74E−10	Down
IKBKE	1.053959	8.04E−05	0.000529	Up
ARSB	1.365099	2.56E−10	8.44E−09	Up
HSPA8	1.498921	9.21E−14	7.24E−12	Up
RGS19	1.765791	1.14E−05	9.97E−05	Up
BIRC5	3.62659	1.86E−05	0.000152	Up

DEARGs, differentially expressed autophagy-related genes; OA, osteoarthritis.

### 3.2 GO and KEGG enrichment analyses of DEARGs

**Figure 4:**
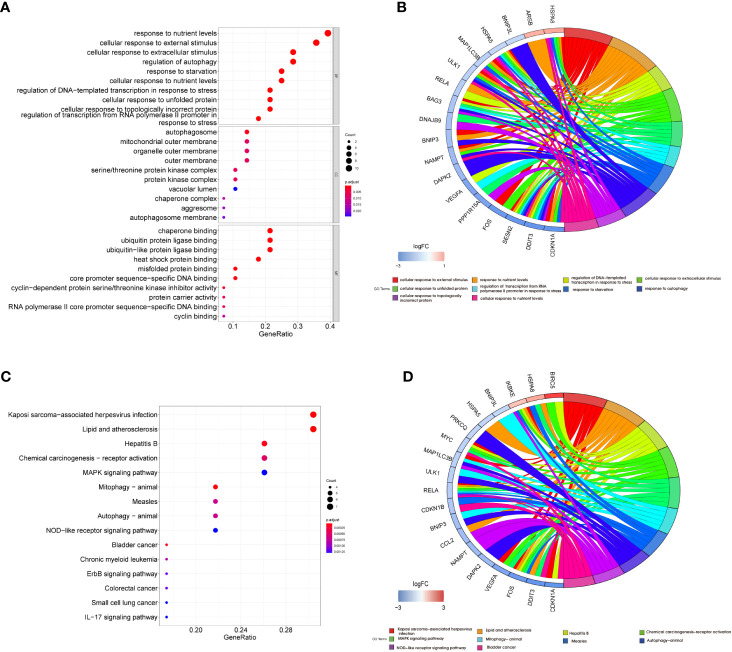
Functional enrichment analysis of differentially expressed autophagy-related genes. Bubble plot **(A)** and Circor chart **(B)** of Gene Ontology (GO) enrichment analysis results of 29 DEARGs in biological process (BP), cellular component (CC), and molecular function (MF). Bubble plot **(C)** and Circor chart **(D)** of KEGG pathway enrichment analysis by DEARGs. DEARGs, differentially expressed autophagy-related genes; KEGG, Kyoto Encyclopedia of Genes and Genomes.

GO and KEGG pathway enrichment analyses were performed on these DEARGs using R software to explore their potential biological functions and pathways. The results of GO functional analysis revealed that the most significant items of GO enrichment included response to nutrient levels, cellular response to external stimulus, cellular response to extracellular stimulus, and regulation of autophagy (biological process); autophagosome, mitochondrial outer membrane, organelle outer membrane, and outer membrane (cellular component); chaperone binding, ubiquitin-protein ligase binding, ubiquitin-like protein ligase binding, and heat shock protein binding (molecular function) (**Figures 4A, B**). The results of KEGG pathway enrichment analysis showed that DEARGs were mainly enriched in autophagy, mitophagy, and inflammation-related pathways (**Figures 4C, D**).

### 3.3 Correlation analysis and PPI network analysis of DEARGs

**Figure 5:**
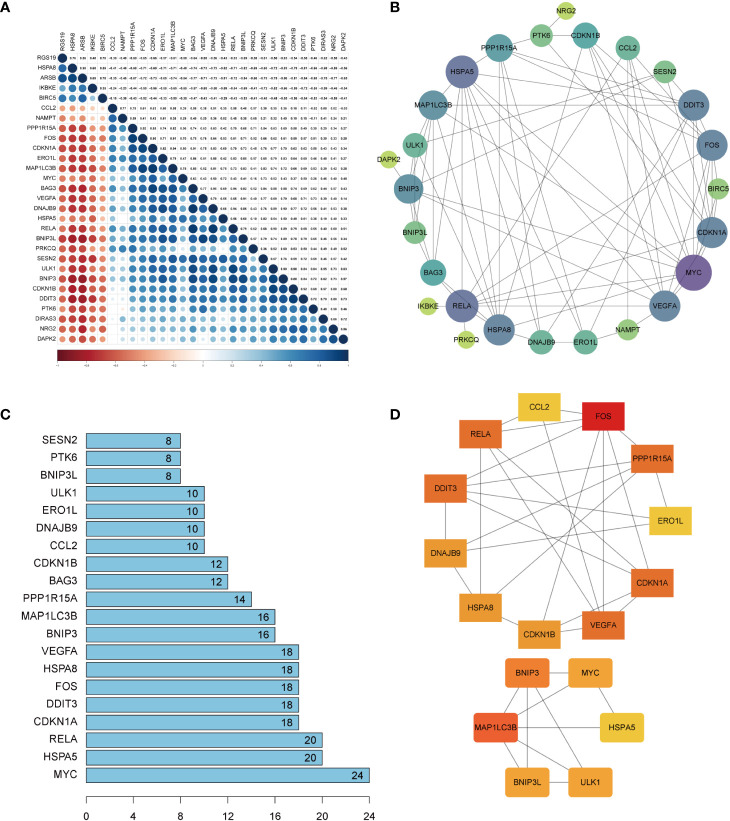
Correction analysis and protein–protein interaction (PPI) analysis of the 29 differentially expressed autophagy-related genes. **(A)** Spearman’s correction analysis of the 29 differentially expressed autophagy-related genes. **(B)** PPI network of DEARGs; three disconnected nodes were removed. Circle size represents the node degree. **(C)** The interaction number of the top 20 DEARGs. **(D)** Two DEARG clusters were obtained using the MCODE plug-ins. DEARGs, differentially expressed autophagy-related genes.

Spearman’s correlation analysis was used to explore the correlation of these DEARGs in OA. The results of the study indicated an interaction between 29 DEARGs (**Figure 5A**). A PPI network analysis was performed to determine the interactions between these DEARGs. PPI networks showed that DEARGs were involved in 26 nodes and 77 edges (**Figure 5B**) and the top 20 interactive genes (**Figure 5C**). According to the MCODE plug-ins of Cytoscape software, two clusters can be obtained (**Figure 5D**). The first cluster consists of 11 genes (FOS, CDKN1A, VEGFA, RELA, DDIT3, PPP1R15A, ERO1L, CDKN1B, DNAJB9, HSPA8, and CCL2), whereas the second cluster involves six genes (BNIP3, MYC, HSPA5, ULK1, BNIP3L, and MAP1LC3B).

**Figure 6:**
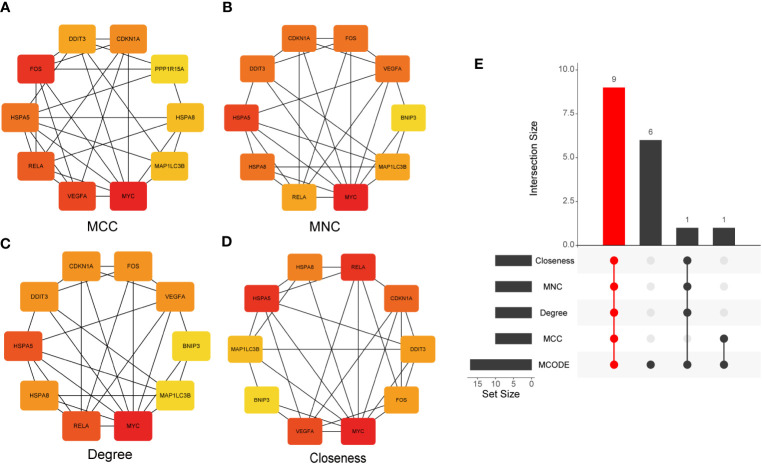
Identification of hub genes. **(A–D)** The PPI network constructed by 26 DEARGs was calculated using four algorithms, namely, MCC, MNC, Degree, and Closeness, and the top 10 genes were selected as hub genes. **(E)** UpSet diagram displays the intersection of five different algorithms, resulting in nine shared hub genes. PPI, protein–protein interaction; DEARGs, differentially expressed autophagy-related genes.

Subsequently, the CytoHubba plug-in was used to calculate the PPI network constructed by the 26 DEARGs using four algorithms (MCC, MNC, Degree, and Closeness) and selected the top 10 genes as hub genes (**Figures 6A–D**). Finally, hub genes obtained by the CytoHubba algorithm were intersected with those calculated using the MCODE algorithm, and nine common hub genes were obtained, which included CDKN1A, DDIT3, FOS, VEGFA, RELA, MAP1LC3B, MYC, HSPA5, and HSPA8 (**Figure 6E**).

### 3.4 Validation of the hub DEARGs by microarray dataset

**Figure 7:**
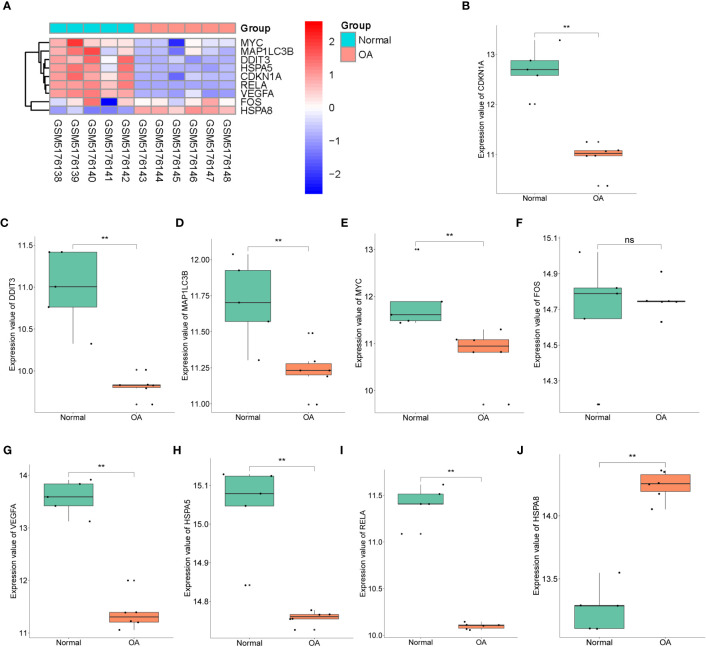
Cross-validation of hub genes. **(A)** The expression levels of *CDKN1A*, *DDIT3*, *FOS*, *VEGFA*, *RELA*, *MAP1LC3B*, *MYC*, *HSPA5*, and *HSPA8* were verified by GSE169077 dataset, the results of which are presented as heatmap. **(B–J)** Detailed expression of nine hub genes. **p < 0.001. ns, not significant.

The expression pattern of hub genes was validated based on the GSE169077 dataset. The heatmap showed the expression levels of nine hub genes (**Figure 7A**). Additionally, as displayed in **Figures 7B–J**, the expression levels of CDKN1A, DDIT3, VEGFA, RELA, MAP1LC3B, MYC, and HSPA5 in OA cartilage were significantly lower than those in control samples, whereas HSPA8 was significantly upregulated (*p* < 0.05), which was in agreement with bioinformatics analysis of GSE114007 dataset. However, no significant difference was found in FOS expression.

### 3.5 Assessment of immune cell infiltration and correlation analysis of hub DEARGs and infiltrating immune cells

**Figure 8:**
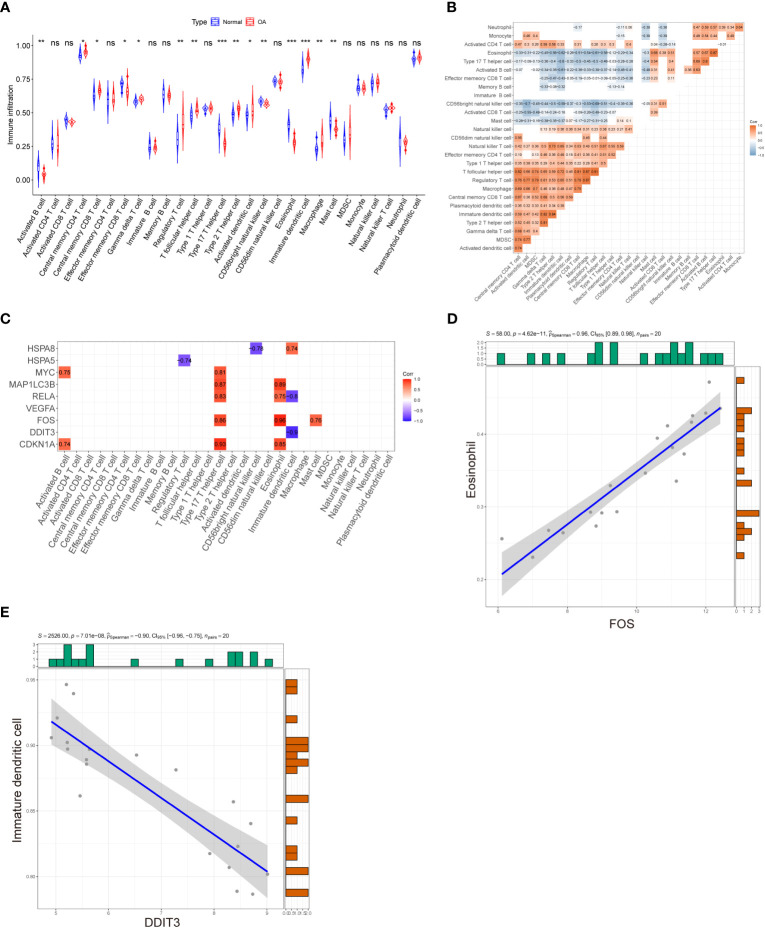
Evaluation of immune cell infiltration and correlation analysis between hub genes and immune cell infiltration. **(A)** Violin plot of differential infiltrating fractions of all 28 immune cells between OA and normal samples. **(B)** Spearman’s correlation heatmap demonstrates the correlation of all 28 immune cells. The blank squares in the upper left corner represent *p*-values >0.05, red presents a positive correlation, and blue denotes a negative correlation. **(C)** Correlation heatmap displaying the correlations between nine hub genes and 28 infiltrating immune cells. The red and purple squares indicate that the hub genes have a significant correlation with infiltrating immune cells, and the blank squares represent *p*-values >0.05 for the association of hub genes with infiltrating immune cells. **(D)** The most positively correlated autophagy–immunocyte gene pair is FOS–Eosinophil. **(E)** The most negatively correlated autophagy–immunocyte gene pair is DDIT3–Immature dendritic cell. *p*-Values were calculated using the Wilcoxon test. ***p < 0.001; **p < 0.01; *p < 0.05. ns, not significant.

ssGSEA was applied to explore immune cell infiltration in the OA cartilage. The results are shown in **Figure 8A**; the proportions of regulatory T cells, activated dendritic cells, central memory CD4 T cells, T follicular helper cell, type 2 helper cell, macrophage, central memory CD8 T cells, gamma delta T cells, and immature dendritic cell were higher in the OA group and lower in the control group. In contrast, the proportions of activated CD56 bright nature killer cells, mast cells, type 17 T helper cells, B cells, eosinophil, and effector memory CD8 T cells were relatively low in the OA group compared to the control group. Subsequently, the correlation of 28 subpopulations of immune cell infiltrate was further analyzed. As shown in **Figure 8B**, except for immature B cells, a significant correlation was found among infiltrating immune cells. Finally, to explore the relationship between hub DEARGs and infiltrating immune cells, Spearman’s correlation analysis was performed (**Figure 8C**). The results suggest that the most positively correlated autophagy–immunocyte gene pair is FOS–Eosinophil, while the most negatively correlated autophagy–immunocyte gene pair is DDIT3–Immature dendritic cell (**Figures 8D, E**).

### 3.6 Validation of hub DEARGs using an *in vitro* OA cell model

**Figure 9:**
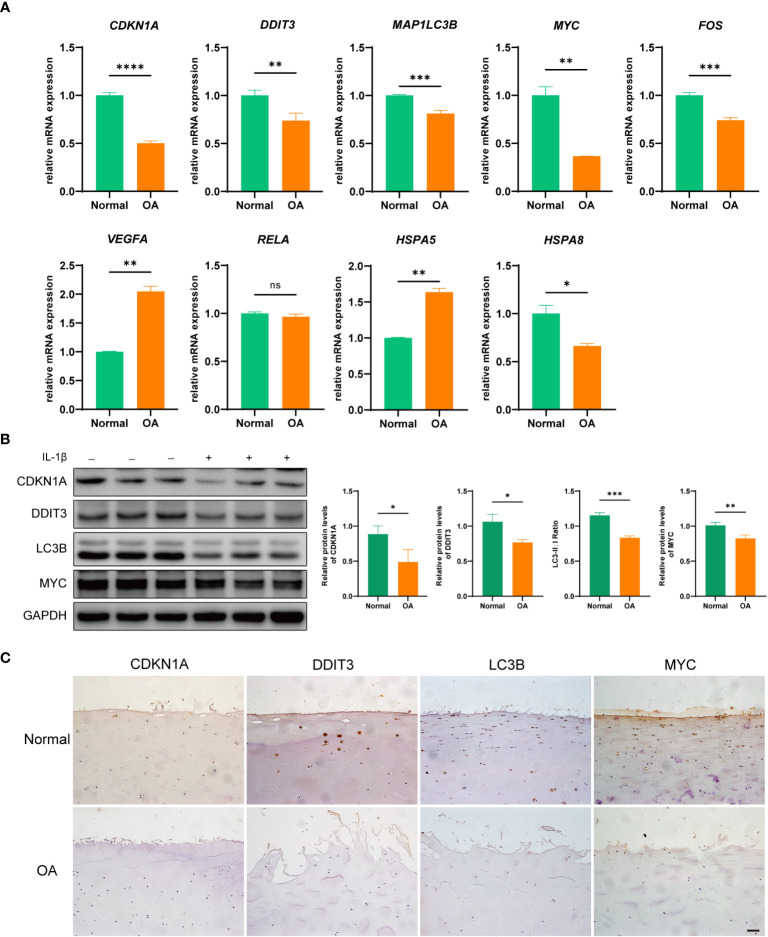
Validation of hub DEARGs using an *in vitro* OA cell model. **(A)** Boxplots show mRNA expression levels of nine hub genes that were measured by qRT-PCR in OA and normal chondrocytes. **(B)** The expression levels of CDKN1A, DDIT3, MAPLC3B, and MYC were detected by Western blotting. **(C)** The expression levels of CDKN1A, DDIT3, MAPLC3B, and MYC were detected by IHC. *p < 0.05, **p < 0.01, ***p < 0.001, and ****p < 0.0001. ns, not significant; DEARGs, differentially expressed autophagy-related genes; OA, osteoarthritis; IHC, immunohistochemistry. Scale bar, 50 μm.

To verify the expression of hub DEARGs in OA chondrocytes, we further performed qRT-PCR and Western blotting. qRT-PCR results showed that the mRNA expressions of *CDKN1A*, *DDIT3*, *FOS*, *MAP1LC3B*, *MYC*, and *HSPA8* were significantly low in the OA group; *HSPA5* and *VEGFA* were significantly highly expressed (*p* < 0.05); and RELA gene expression was not significantly changed compared to normal controls (**Figure 9A**). Moreover, Western blotting results showed that the expression levels of CDKN1A, DDIT3, MAP1LC3B, and MYC were significantly decreased in the OA group (**Figure 9B**). Further, the IHC staining showed that compared with the normal group, expressions of CDKN1A, DDIT3, MAP1LC3B, and MYC were low in the OA articular cartilage tissue (**Figure 9C**).

## 4 Discussion

OA is the most common chronic degenerative joint disease with limited treatment options, as its etiology has not been fully elucidated (4). Many studies in recent years have demonstrated that autophagy defects contribute to OA and age-related diseases; in contrast, autophagy activation could promote cell survival and reduce the severity of experimental OA (24, 25). However, the relationship between autophagy and OA is currently not fully understood. In addition, ARGs in OA cartilage have not been well explored using bioinformatics analysis. Therefore, we conducted this study to explore whether the expression of ARGs in OA cartilage tissue was significantly different from that in healthy controls. Additionally, we investigated the correlation between ARGs and immune infiltration, which can help understand the potential immune mechanisms of ARGs in cartilage and identify potential molecular targets in OA therapy.

In this study, bioinformatics analysis tools were used to identify ARGs and 29 potential DEARGs associated with OA. Among them, five genes were found to be upregulated in expression and 24 genes were downregulated in expression, as shown in **Table 2**. After construction using the PPI network, nine key ARGs (*CDKN1A*, *DDIT3*, *FOS*, *VEGFA*, *RELA*, *MAP1LC3B*, *MYC*, *HSPA5*, and *HSPA8*) were differentially expressed in OA cartilage. To understand the potential molecular biological function of DEARGs between OA and normal cartilage, we performed enrichment analysis using GO and KEGG. The enrichment results suggested that these DEARGs play important roles in various biological functions, including autophagy, mitochondrial autophagy, and inflammation-related pathways. In this case, we collected clinical samples to validate the expression of these nine genes. *In vitro*, cellular experiments showed that the expression of four genes, *CDKN1A*, *DDIT3*, *MAP1LC3B*, and *MYC*, were significantly consistent (*p* < 0.05) in bioinformatics analysis. Some ARGs have been previously studied in OA. *CDKN1A* is also known as protein p21. It was shown that *CDKN1A* expression was decreased in OA by inhibiting chondrocyte proliferation (26). Moreover, Huang et al. revealed that in cardiomyocytes, autophagy was inhibited when the expression level of p21 was decreased and that p21 regulates autophagy by interacting with *MAP1LC3B(LC3B)* (27). Yang et al. showed that *DDIT3* knockdown suppressed the autophagy of chondrocytes *in vitro* and *in vivo*, causing a decrease in *LC3B* and *Beclin1* gene expression; however, overexpression of *DDIT3* significantly promoted autophagy *in vitro* (28). In microglia, DEX promotes NLRP3 inflammasome degradation via the autophagy-ubiquitin pathway and reduces *MAP1LC3B* expression, thereby reducing hippocampal inflammation (29). Anti-Dlx5 slows the progression of OA by downregulating the chondrocyte apoptosis-related gene *MYC* (30). These results are consistent with our bioinformatics analysis and experimental results. Moreover, four genes, namely, *CDKN1A*, *DDIT3*, *MAP1LC3B*, and *MYC*, may determine the progression of OA. The specific mechanisms need to be further investigated.

Many publications have reported that the infiltration of immune cells is a key factor in promoting the development of OA (31). For example, immune cells such as neutrophils, M1 macrophages, CD4+ T cells, and mast cells have a significant infiltration in OA synovium, suggesting that immune infiltration is a key target for the treatment of osteoarthritis (31–33). Many immune infiltration studies have been performed in OA synovial tissue, and relatively few immune infiltration studies have been performed in cartilage tissue. Therefore, we comprehensively evaluated the types of immune infiltration cells in OA cartilage tissues using ssGSEA, hoping to explore in-depth the potential mechanisms of immune infiltration in OA cartilage. We performed a correlation analysis of immune cell subsets in normal cartilage tissue and OA samples. It was found that the proportions of the 28 immune cell subpopulations differed significantly between healthy cartilage and OA cartilage samples. The proportion of regulatory T cells, activated dendritic cells, central memory CD4 T cells, T follicular helper cells, type 2 helper cells, macrophage, central memory CD8 T cells, gamma delta T cells, and immature dendritic cells were present in a higher proportion of OA samples compared with healthy controls. In contrast, the fraction of activated CD56 bright nature killer cells, mast cells, type 17 T helper cells, B cells, eosinophils, and effector memory CD8 T cells was lower in OA. Han et al. performed analysis using ssGSEA, MCPcounter, and ESTIMATE software and found that immune scores were significantly lower in normal control samples than in OA samples (34). The discrepancy could be caused by the difference in the methods of analysis and sample numbers. Previous studies have illustrated that the immune system is associated with autophagic pathways, including innate and adaptive immunity, and when autophagy is disturbed, it contributes to the implications of inflammatory diseases (35–37). However, few studies have shown the relationships between ARGs and immune cell infiltration in OA cartilage.

This study also analyzed the potential link between nine key ARGs and infiltrating immune cell subpopulations, contributing to an in-depth understanding of the regulatory role of the ARGs in OA cartilage and also providing new insights into the pathogenesis of OA. Our study showed that *DDIT3* and *FOS* genes were significantly correlated with immune cells, with *FOS* expression being positively associated with eosinophils and *DDIT3* expression being positively associated with immature dendritic cells. It has been reported that *FOS* may treat dermatomyositis by regulating the infiltration of immune cells (38). Deng et al. found that infiltration of eosinophils may be associated with OA progression (32). Dendritic cells (DCs) present a prominent immunomodulatory capacity in innate and adaptive immune responses and are specialized antigen-presenting cells. DCs in OA patients have been reported to secrete large amounts of inflammatory cytokines that exacerbate the inflammatory response (39). However, no report proved the role of eosinophils in OA; in addition, the pairs *FOS*–Eosinophil and *DDIT3*–Immature dendritic cell have also not been investigated in OA. These findings suggest that there may be an interaction between autophagy and the immune response in OA. All of these studies suggest that autophagy and immune infiltration may have a reciprocal regulatory role in OA cartilage. However, more experimental studies may be needed to elucidate in-depth the mechanisms of these ARG–immune cell interactions for validation.

However, this study still has some inevitable limitations. First, we re-mined and analyzed previously published datasets. Second, not multiple datasets but only one dataset was used for cross-validation, and datasets and clinical samples were insufficient in our study, which may lead to deviations in the results. Thus, larger sample sizes are required to confirm our findings. Finally, we simply verified the expression levels of hub DEARGs by qRT-PCR, Western blotting, and IHC staining *in vitro* by collecting clinical samples without in-depth exploration of the underlying mechanism. Therefore, further studies need to be conducted to reveal the underlying molecular mechanisms.

## 5 Conclusions

In conclusion, we identified nine key ARGs, namely, CDKN1A, DDIT3, FOS, VEGFA, RELA, MAP1LC3B, MYC, HSPA5, and HSPA8, in OA cartilage tissue by informatics analysis. Verified results confirm that CDKN1A, DDIT3, MAP1LC3B, and MYC could serve as potential biomarkers for OA cartilage tissue. Additionally, the correlation analysis between hub ARGs and immune infiltration in OA cartilage suggested that the interaction between ARGs and immune cell infiltration may be involved in regulating the pathogenesis of OA. Among them, the expression level of DDIT3 showed a strong negative correlation with immature dendritic cells. The present study revealed that autophagy may regulate the progression of OA by regulating immune infiltration, providing new insights into the molecular immune mechanisms of treating OA.

## Data availability statement

The original contributions presented in the study are included in the article/**Supplementary Material**, further inquiries can be directed to the corresponding author/s.

## Ethics statement

The studies involving humans were approved by The Ethics Committee of Guangxi Medical University (NO. 2019-SB-058, 7 Mar. 2019). The studies were conducted in accordance with the local legislation and institutional requirements. Written informed consent for participation in this study was provided by the participants’ legal guardians/next of kin.

## Author contributions

JQ: Conceptualization, Data curation, Formal Analysis, Methodology, Resources, Software, Validation, Visualization, Writing – original draft, Writing – review & editing. JZ: Conceptualization, Data curation, Methodology, Resources, Validation, Writing – original draft. J-JW: Conceptualization, Data curation, Software, Validation, Visualization, Writing – review & editing. XR: Data curation, Investigation, Writing – original draft. Q-LZ: Investigation, Writing – original draft. J-MZ: Funding acquisition, Project administration, Supervision, Writing – review & editing. N-HL: Funding acquisition, Project administration, Supervision, Writing – review & editing.
