# Supplementary material for: Identification of autophagy-related genes in osteoarthritis articular cartilage and their roles in immune infiltration
Source: Front Immunol. 2023 Nov 27;14:1263988. doi: 10.3389/fimmu.2023.1263988 (PMC10711085; doi:10.3389/fimmu.2023.1263988)
Supplement: Supplementary file 1 [file DataSheet_1.docx]

Supplementary Material

# Supplementary Figures


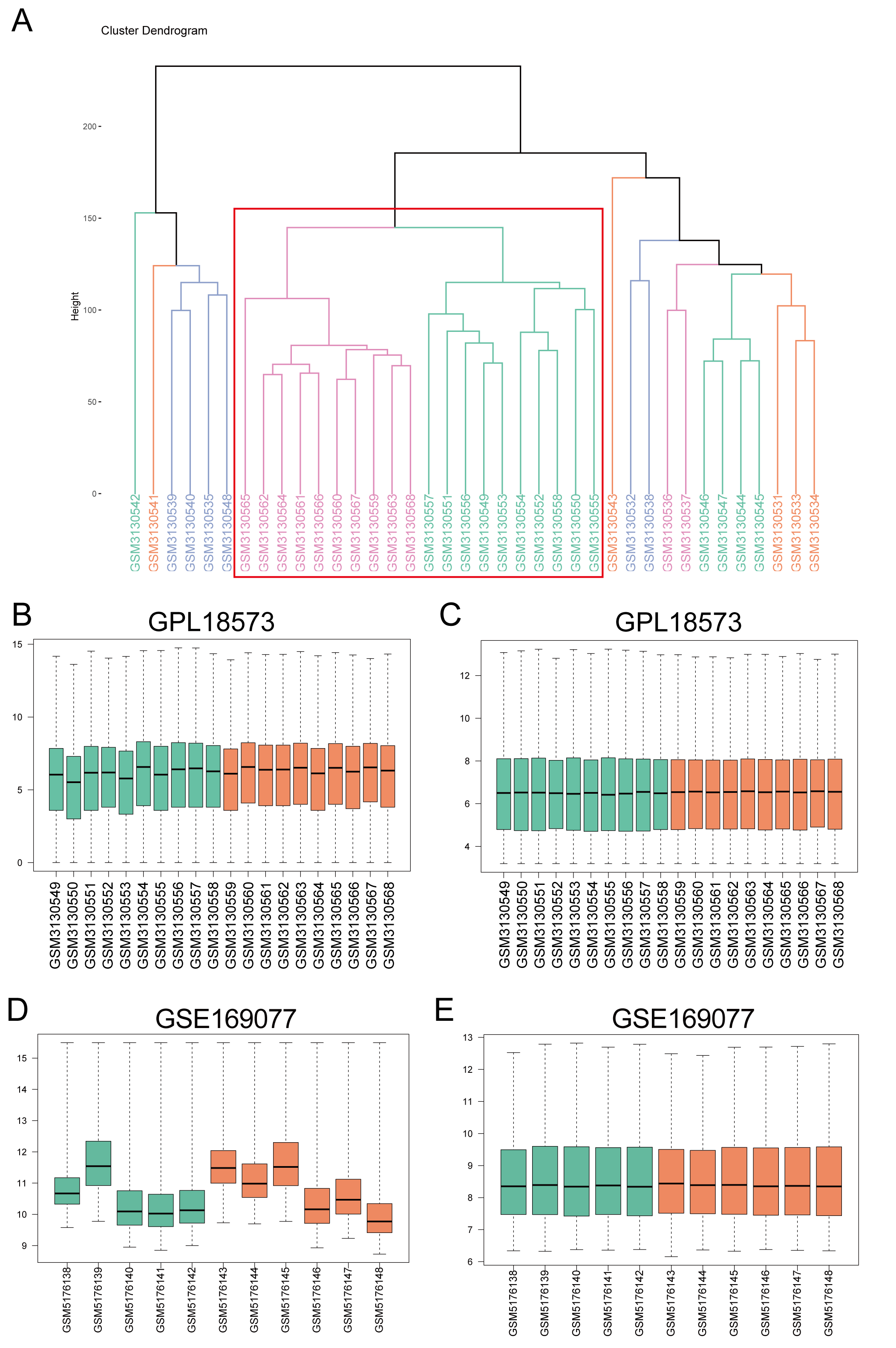


**Supplementary Figure S1.** Quality assessment of GSE114007 dataset. **(A)** Cluster dendrogram of GSE114007. The cluster in the red box represents GPL18573. **(B-C)** Boxplots of GSE114007 data set prior to and following normalization. **(D-E)** Boxplots of GSE169077 data set prior to and following normalization.

**Table S1** | The detailed information of the GEO datasets used for analysis

| **GEO Dataset** | **Platform** | **Species** | **Tissue** | **OA** | **Normal** |
| --- | --- | --- | --- | --- | --- |
| GSE114007 | GPL18573 | Homo sapiens | Knee cartilage | 10 | 10 |
|  | GPL11154 | Homo sapiens | Knee cartilage | 10 | 8 |
| GSE169077 | GPL96 | Homo sapiens | Knee cartilage | 6 | 5 |


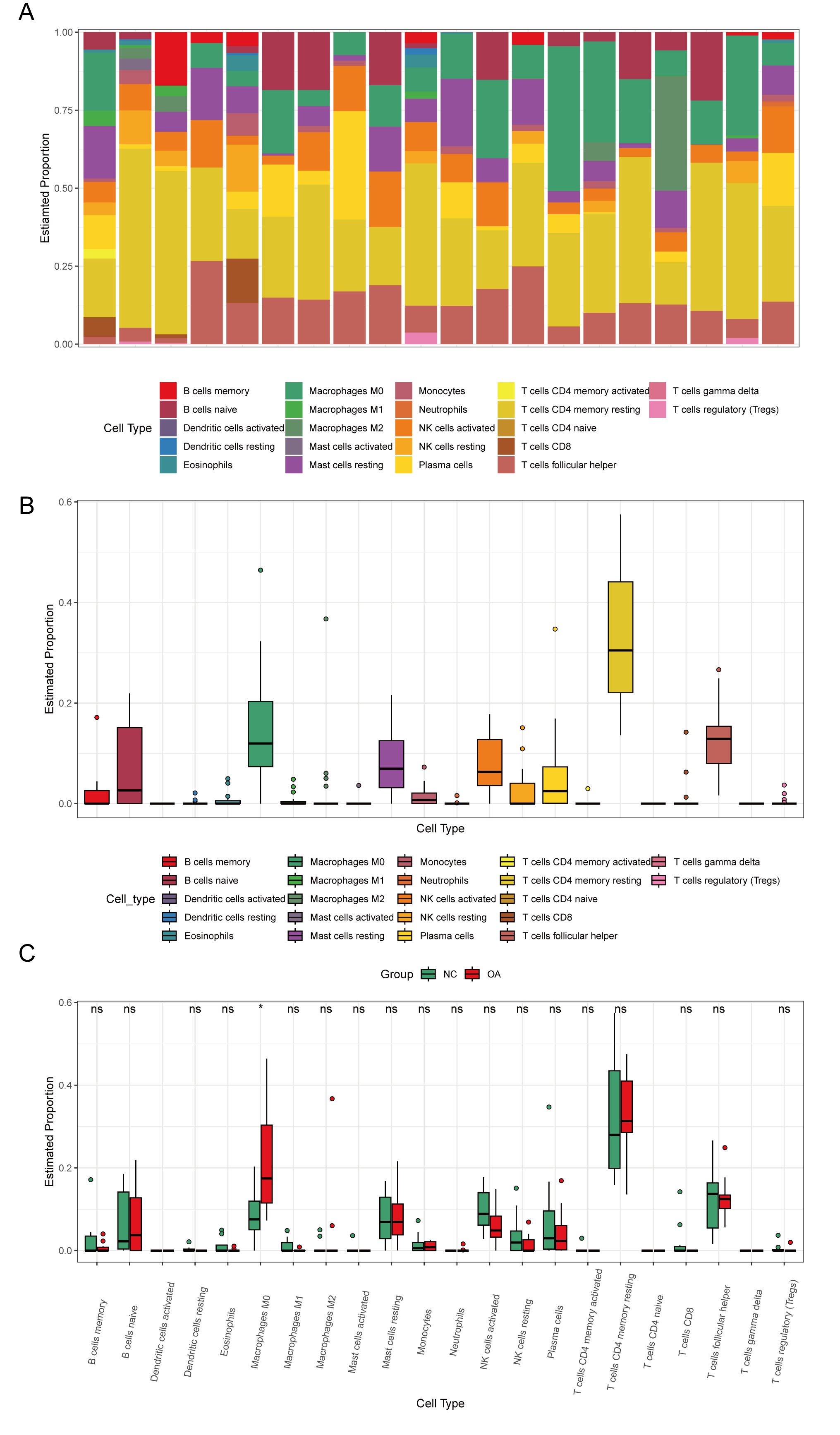


**Supplementary Figure S2.** CIBERSORT algorithm was adopted to investigate the immune-cell infiltration. **(A)** The stacked bar chart shows the distribution of 22 immune cells in each sample. **(B)** The proportion of 22 types of immune cells. **(C)** Boxplots of differential infiltrating fractions of all 22 immune cells between OA and normal samples.
